# Effect of the COVID-19 Pandemic on the Pediatric Emergency Department Flow

**DOI:** 10.1017/dmp.2021.355

**Published:** 2021-12-20

**Authors:** Andrea Rivera-Sepulveda, Timothy Maul, Katherine Dong, Kylee Crate, Talia Helman, Corinne Bria, Lisa Martin, Kimberly Bogers, Joseph W. Pearce, Todd F. Glass

**Affiliations:** 1 Department of Pediatrics, Division of Emergency Medicine, Nemours Children’s Hospital, Orlando, Florida, USA; 2 Department of Cardiac Surgery, Nemours Children’s Hospital, Orlando, Florida, USA; 3 University of Pittsburgh, Department of Bioengineering, Pittsburgh, Pennsylvania, USA; 4 University of Central Florida College of Medicine, Orlando, Florida, USA; 5 Department of Emergency Services, Nemours Children’s Hospital, Orlando, Florida, USA

**Keywords:** COVID-19, coronavirus, pediatric emergency department, use, flow

## Abstract

**Objective:**

The aim of this study was to determine how the early stages of the coronavirus disease 2019 (COVID-19) pandemic affected the use of the pediatric emergency department (PED).

**Methods:**

Cross-sectional study of PED visits during January through April, 2016-2020. Data included: total PED visits, emergency severity index (ESI), disposition, chief complaint, age (months), time from first provider to disposition (PTD), and PED length of stay (PED-LOS). *P*-value <0.01 was statistically significant.

**Results:**

In total, 67,499 visits were reported. There was a significant decrease in PED visits of 24-71% from March to April 2020. Chief complaints for fever and cough were highest in March 2020; while April 2020 had a shorter mean PED-LOS (from 158 to 123 min), an increase of admissions (from 8% to 14%), a decrease in ESI 4 (10%), and an increase in ESI 3 (8%) (*P* < 0.001). There was no difference in mean monthly PTD time.

**Conclusions:**

Patient flow in the PED was negatively affected by a decrease in PED visits and increase in admission rate that may be related to higher acuity. By understanding the interaction between hospital processes on PEDs and patient factors during a pandemic, we are able to anticipate and better allocate future resources.

The SARS-coronavirus-2 virus causes an acute respiratory disease (coronavirus disease 2019 [COVID-19]) that has posed great challenges to the health-care system due to its widespread global incidence and increasing number of cases. As of April 30, 2020, there were 3,090,445 laboratory-confirmed COVID-19 cases worldwide, and 217,769 deaths.^1^ While in the United States as of the same date, there were 1,031,659 total cases reported in the United States and 60,057 deaths^2^; with Florida reporting 32,318 cases with 1218 deaths.^3^ The <18-y age group had 16,980 cases, accounting for 2% of total cases in the United States. In the beginning of the COVID-19 pandemic, the pediatric population appeared to have the lowest rate of infection for all age groups.^4^ More recent data by the Centers for Disease Control and Prevention (CDC), in May 2021, suggest that the number and rate of COVID-19 cases in children have been steadily increasing, with some developing more severe disease.^5^

The last widespread occurrence of an infectious disease to affect the pediatric population in the United States was the influenza A (H1N1) virus in 2009. During the 2009 influenza A epidemic, studies demonstrated a relative increase in emergency department (ED) visits^6^ and showed that hospitals routinely operated close to maximum capacity, with little available reserve even for a modest surge in patients.^7^ However, there is limited research on pandemics and how they impact hospital systems and medical resources. Because surge capacity may be scarce in pediatric hospitals throughout the United States, it is important to understand how the pediatric population uses the pediatric ED (PED) and its resources in times of public health emergencies. Therefore, the aims of this study are to: (1) determine the rate of PED visits during the COVID-19 pandemic, and compare with the 2017-2018 influenza epidemic; (2) determine the types of cases that presented to the PED during the COVID-19 pandemic; and (3) correlate the rate of PED visits with the level of acuity.

## Methods

### Study Design

This is a retrospective cross-sectional study of patients who visited the PED at a specialty-based, free-standing children’s hospital from January 1 to April 30 during the years 2016 through 2020.

### Data Source

**Figure 1.**
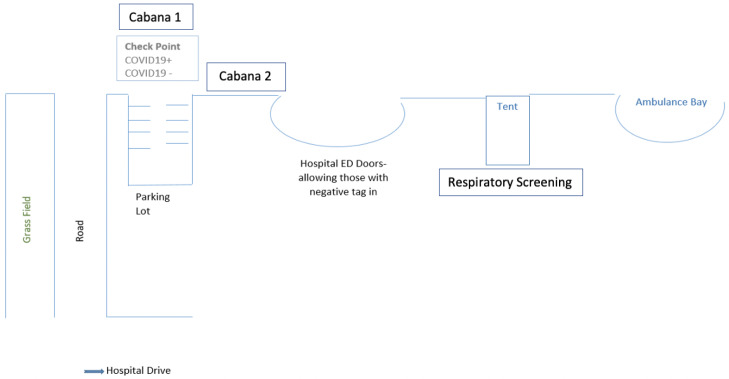
COVID-19 tent screening organization. Screening occurs before arrival to the ED parking lot (Cabana 1). Respiratory cases with positive screens are directed to stay in their cars and undergo triage and intake assessment through the external drive-through tent. Critically ill, high-risk patients, and those with negative screens are directed to park and proceed to have the parent/guardian screened (Cabana 2) and enter through the main ED.

The de-identified data were provided as a single line per visit by QlikView (QlikTech Inc., PA), a business intelligence (BI) data discovery product used for creating guided analytic applications and dashboards tailor-made to generate data. The data included in this study are based on quality metrics used as part of the standard of care within the PED. Our study was based on a tertiary, freestanding, university-affiliated, children’s community hospital with a 2.1 case mix index (CMI). The hospital consists of 100 beds, a medical-surgical unit, and pediatric, neonatal, and cardiac intensive care units. The PED receives approximately 40,000 visits annually, and is staffed by 25 providers, comprising 19 full-time equivalents (47% pediatric emergency medicine, 16% medical doctor (MD)/general pediatrician, 37% advanced provider) and 45-50 registered nurses and medics/paramedics. A tent screening process was developed and implemented as part of the hospital operational changes to manage a COVID-19 surge from March 23 to April 30, 2020. The screening process involved the strategic placement of external drive-through tents for triage and intake assessments to mitigate potential exposure to infectious diseases (Figure 1).

### Study Variables

Data included date of PED visit (daily census), age (in months), emergency severity index (ESI), primary diagnosis and secondary diagnosis based on the *International Classification of Diseases, Tenth Revision* (ICD-10) code, chief complaint, and disposition. Disposition included the allocation of admission, discharge, death, left without treatment, and transfer. Admissions were further stratified by location onto inpatient floor, surgery unit, or intensive care unit (ICU). The ICU combined the pediatric, neonatal, and cardiac intensive care units. PED flow time was reported in minutes as: door to triage, door to bed, door to first provider, first provider to disposition (PTD), disposition order to depart, and overall PED length of stay (PED-LOS). We performed a sub-group analysis of the PED flow metrics on the PED tent system. The ESI was defined based on the 5-level ED triage algorithm that provides clinically relevant stratification of patients into 5 groups from 1 (most urgent) to 5 (least urgent) on the basis of acuity and resource needs.^8^ PED visits were codified based on the primary diagnosis upon disposition into the following categories: (1) neurologic (ie, altered mental status, seizure), (2) respiratory (ie, asthma, bronchiolitis, pneumonia, respiratory distress, influenza), (3) gastrointestinal (ie, Crohn’s disease, ulcerative colitis, abdominal pain, vomiting, diarrhea), (4) endocrine (ie, diabetic ketoacidosis, hypoglycemia), (5) orthopedic (ie, fracture, dislocation), (6) injury (ie, closed head injury, contusion, laceration), (7) hematology/oncology (ie, sickle cell fever/pain, fever, and neutropenia), (8) infectious (ie, acute febrile illness, viral illness/syndrome, upper respiratory tract infection, urinary tract infection, cellulitis), (9) surgical (ie, intussusception, appendicitis, testicular/ovarian torsion, pyloric stenosis), and (10) not otherwise specified (NOS) (ie, behavioral, rash, intoxication).

### Outcome Measures

The primary outcome measures were: (1) PED daily visit rate per month and year, (2) PED daily visit rate during the COVID-19 pandemic season compared with the 2017-2018 influenza epidemic, (3) distribution of cases during the study period, and (4) correlation of PED visit rate with the ESI. Other outcomes of interest included disposition, length of stay in the PED, and temporal distribution of PED visit rate in relation to the national and state pandemic mandates by the CDC and the US government.

### Statistical Analysis

A descriptive analysis of data from patients who visited the PED from January through April 2016-2020 was done using parametric and nonparametric techniques. Continuous variables were described using means and standard deviations (SDs), and categorical variables were described using counts and percentages. Extreme outliers for continuous variables were defined as values that were more than 3 times the interquartile range beyond the 75 percentile and were removed from further analysis. The raw data were extracted into separate columns for day, month, and year. Aggregation of the data was performed for each day in a given year (1 through 366). Total daily PED visits, mean age, PED flow metrics, and percentage of admissions for the same month were averaged from 2016 to 2019 and compared with the same month in 2020 using 1-way analysis of variance (ANOVA). ESI was treated as an ordinal categorical variable and was analyzed between the study periods (2016-2019 vs 2020) using gamma testing. We performed an analysis between the year 2018 and 2020, to compare the effect of an unusually high infectious respiratory illness burden, such as the 2017-2018 influenza epidemic, with the COVID-19 pandemic on PED visits. For further analysis of the distribution of cases based on their categorical diagnoses and chief complaints, we identified the categories/complaints that significantly deviated from the expected distribution by adjusted z-values, and getting a *P*-value for a 1 degree of freedom on a chi-squared with a Bonferroni correction. All statistical analyses were conducted in IBM SPSS Statistics 25 (IBM Corp., Armonk, NY) with *P*-values less than 0.01 considered statistically significant. A descriptive analysis was performed to evaluate the association between total daily census of PED visits and the implementation of the national and state pandemic mandates by the CDC and the US government. This study was approved by the Office of Human Subjects Protection.

## Results

### PED Visit Rate Per Month and Year

A total of 67,499 visits were reported for the months of January through April from 2016 to 2020 (Table 1). Our study showed a decrease in PED visits of 24% in late March (coinciding with the Florida closure of educational facilities) and 71% in April 2020 compared with the same period a year earlier. The mean PED daily census decreased from 118, SD 21 in 2016-2019 to 88, SD 40 in 2020 (*P* < 0.001). We reported a decreasing trend in the daily PED census for the months of February (from 131, SD 22 to 114, SD 20), March (from 116, SD 14 to 88, SD 34), and April (115, SD 14 to 33, SD 5) in 2020 when compared with the same time period in prior years (*P* < 0.001). Patients who were ≥18 y of age accounted for 1.1% of total PED visits. We found no significant difference in age between study periods.

**Table 1.** Comparison of selected characteristics of PED visits during the study period

Mean ± SD (SEM) or *N* (%)	Study period		*P*-Value
	2016-2019 (*N* = 56,843)	2020 (*N* = 10,656)	
PED daily census	118 ± 21 (0.96)	88 ± 40 (3.6)	0.000
Age (months)	76 ± 61 (0.26)	76 ± 62 (0.60)	0.846
ESI			
1	17 (0.03)	12 (0.1)	0.000
2	3,459 (6)	578 (6)	
3	17,915 (32)	3,264 (31)	
4	30,307 (54)	5,532 (52)	
5	4,609 (8)	1,155 (11)	
Disposition			
Admission	4,456 (8)	891 (8)	0.05
Discharge	52,221 (92)	9,727 (91)	
Death	0 (0)	1 (0.01)	
Left without treatment	10 (0.02)	2 (0.01)	
Transfer	156 (0.3)	35 (0.3)	
Admission location			
Inpatient floor	3,674 (81)	728 (81)	0.02
Surgery unit	277 (6)	74 (8)	
ICU	588 (13)	98 (11)	

Abbreviations: ESI, emergency severity index; ICU, intensive care unit; PED, pediatric emergency medicineI; SD, Standard deviation; SEM, standard error of the mean.

### Comparison of the PED Visit Rate During the COVID-19 Pandemic Season With the 2017-2018 Influenza Epidemic

In 2018, 15,478 PED visits were reported, with an increase (*P* < 0.001) in the mean daily census in January (135%) and February (113%) compared with the mean daily census for these months in 2016, 2017, and 2019. This finding coincided with an unusually high influenza season burden. This trend, however, disappeared in March and April as the mean daily census in those months became equal in 2018 compared with 2016, 2017, and 2019. In contrast, 2020 had a higher mean daily census in January (117, SD 17; *P* < 0.001) compared with 2016, 2017, and 2019 (103, SD 16), but was lower than 2018 (140, SD 33). From February onward, the mean daily census for 2020 continued to be significantly lower than both 2018 and 2016, 2017, and 2019. Comparing the year 2020 to 2018 on a week-to-week basis, mean daily census for weeks 4, 5, and 13-18 were higher (*P* < 0.003) when using Bonferroni correction. The combination of primary diagnoses for cough, fever, influenza, and influenza-like illness by month for 2018 vs 2020 showed a difference for the months of February (from 13% to 9.3%) and March (from 4.3% to 7.0%) (*P* < 0.001).

### Distribution of Cases During the Study Period Based on Chief Complaint and Primary Diagnosis

The 10 most common chief complaints were fever, abdominal pain, vomiting, cough, breathing problem, ear pain, rash, lower extremity injury, upper extremity injury, and closed head injury without loss of consciousness. The month of January showed an increase in fever complaints (from 22% to 25%; *P* < 0.001), while February had a decrease in cough complaints (from 12% to 10%; *P* < 0.001). The month of March showed an increase in complaints of fever (from 21% to 24%) and cough (from 10% to 16%), with a decrease in complaints of abdominal pain (from 6.6% to 4.5%) and vomiting (from 9.3% to 6.0%) (*P* < 0.001). Moreover, April showed a further decrease in vomiting complaints (from 8.8% to 4.8%; *P* < 0.001).

The distribution of primary diagnosis upon discharge showed that within any given month, there were shifts of any 1 type of category but no overarching trend visible. The month of March (2016-2019 vs 2020) showed that there was a decrease in gastrointestinal cases (from 18% to 13%), and an increase in infectious cases (from 36% to 40%) (*P* < 0.001). April (2016-2019 vs 2020) showed a decrease in respiratory cases (from 11% to 5.7%) and an increase in the categories of injuries (from 8.6% to 12%) and NOS (from 15% to 19%) (*P* < 0.001). The categories for neurologic, endocrine, orthopedic, hematology/oncology, and surgical cases was nonsignificant for the months of March and April across study periods. Sub-group analysis upon the combination of primary diagnosis between influenza-like illnesses (including all fever, cough, influenza, and influenza-like illness cases), compared with non–influenza-like illnesses (rest of primary diagnosis), showed significant differences (*P* < 0.01) between study periods in January and February. During 2020, January had a higher rate of influenza-like illness diagnoses (14% vs 8.5%), while February had a decreasing rate of influenza-like illness diagnoses (9.3% vs 11%).

### Correlation Between the PED Visit Rate and the ESI

Changes in PED visits based on ESI were significant between 2016-2019 and 2020 (*P* < 0.001), but with very low correlation (γ = 0.05) (Table 1). There was a slight increase in relative rate of ESI 5 (2.8%) coupled with drops in ESI 2 (0.7%), 3 (0.9%), and 4 (1.3%) in 2020 compared with 2016-2019. Sub-group analysis of ESI per month showed a decrease in ESI 4-5 on April 2020, but there were no statistical differences in the mean percentage of ESI 1-2 or ESI 3-5 between study periods. Comparison between 2020 versus 2016-2019 showed that patients were more likely to be admitted to ICU when classified as ESI 2 (45% vs 38%; *P* < 0.005) or 3 (17% vs 15%; *P* < 0.01).

### Disposition From the PED

The overall discharge rate from the PED to home was 92%, while 7.9% were admitted to the hospital. The distribution of disposition and admission location between study periods was nonsignificant (*P* < 0.05) (Table 1). However, sub-group analysis showed that the percentage of patients admitted was slightly higher in February (from 7.2% to 8.6%; *P* < 0.01) but sharply rose in April (from 8.2% to 14%, *P* < 0.001) across study periods. In 2020, the months of January and March showed that the surgery unit had an increase in admissions (from 5.8% to 10%; *P* < 0.05; and from 6.4% to 10%; *P* < 0.01, respectively), but a decrease in April from 6.7% to 4.4%. There was also a subsequent decrease in ICU admissions by 2.1% in January and 6.3% in March. In the month of April (2020 vs 2016-2019), admissions to the inpatient floor and ICU increased by 1.6% and 0.7%, respectively. A secondary analysis of surgical admissions treated as a binary variable (ie, surgery vs combination of inpatient floor and ICU) did not change the significance of the distribution of dispositions.

### LOS in the PED

Mean PED-LOS was shorter in 2020 for March (from 156, SD 20 to 128, SD 29 min) and April (from 158, SD 24 to 123, SD 21 min) (*P* < 0.001) (Table 2). First PTD times were longer in February (*P* < 0.01) and April 2020 (*P* = 0.01), but shorter in March (*P* < 0.05). However, as a proportion of PED-LOS, only April was significantly larger (62%, SD 6% vs 50%, SD 6%; *P* < 0.001) between 2020 and 2016-2019. A scatter-plot comparison of the nonaggregated data set for the first PTD compared with PED-LOS showed a shift in the slope for the study periods 2016-2019 and 2020; hence, the first PTD time drives the decrease in PED-LOS. The ratio for all months in the analysis period was significant (*P* < 0.0001) for the same phenomenon.

**Table 2.** Comparison of the flow metrics within the Pediatric Emergency Department during the study periods

Mean ± SD (SEM)	Jan – Feb			Mar – Apr		
	2016-2019 (N = 28,716)	2020 (N = 6,937)	*P*-Value	2016-2019 (N = 28,127)	2020 (N = 3,719)	*P*-Value
Door to bed	39 ± 43 (0.26)	41 ± 41 (0.49)	0.001	35 ± 38 (0.23)	21 ± 29 (0.48)	0.000
Door to first provider	55 ± 48 (0.29)	51 ± 44 (0.53)	0.000	51 ± 43 (0.26)	30 ± 33 (0.55)	0.000
PTD	79 ± 60 (0.37)	77 ± 65 (.81)	0.035	80 ± 61 (0.38)	75 ± 66 (1.1)	0.000
Disposition order to depart	22 ± 16 (0.1)	20 ± 15 (0.20)	0.000	22 ± 16 (0.10)	20 ± 15 (0.30)*	0.000
PED-LOS	163 ± 86 (0.51)	155 ± 88 (1.1)	0.000	158 ± 85 (0.51)	133 ± 90 (1.5)	0.000

Abbreviations: PED-LOS, pediatric emergency department length of stay; PTD, first provider to disposition; SD, standard deviation; SEM, standard error of the mean.

* Only available through March 29, 2020, due to use of outdoor tent system.

### COVID-19 Tent Screening Process

There were 490 patient encounters made in the PED tent system from March 23 to April 30, 2020 (Table 3). Seventy-eight percent (78%) of patients were seen and discharged from the PED tent setting. Sub-group analysis performed solely on the encounters made through the PED tent system was significant for PED-LOS, door to triage, first PTD, and disposition order to depart (all *P* < 0.001). The mean PED-LOS for patients seen in the tent system was 40 min (SD 49 min). For those patients who were transferred from the PED tent to the PED (22%), the PED-LOS increased to 193 min (SD 107 min, *P* < 0.001). There was no difference in the rate of return within 48 h (3.0% for both groups, *P* = 1.0) between the discharges from the PED tent system, and those discharged from the PED.

**Table 3.** Comparison of the flow metrics within the PED based on the primary site of evaluation during the implementation of the PED tent system

Mean ± SD (SEM)	PED tent system (*N* = 383)	Transfer from PED tent to PED (*N* = 107)	*P*-Value
Door to triage start	6 ± 5 (0.28)	7 ± 6 (0.63)	0.006
Door to bed	4 ± 4 (0.19)	4 ± 4 (0.38)	0.636
Door to first provider	13 ± 11 (0.57)	14 ± 14 (1.3)	0.265
PTD	21 ± 41 (2.2)	133 + 80 (8.4)	0.000
Disposition order to depart	12 ± 10 (1.0)	29 ± 20 (5.5)	0.000
PED-LOS	40 ± 49 (2.49)	193 ± 107 (10.5)	0.000

Abbreviations: PED-LOS, pediatric emergency department length of stay; PTD, first provider to disposition; SD, standard deviation; SEM, standard error of the mean.

### Temporal Distribution of Daily PED Visit Rate in Relation to the National and State Pandemic Mandates by the CDC and the US Government

**Figure 2.**
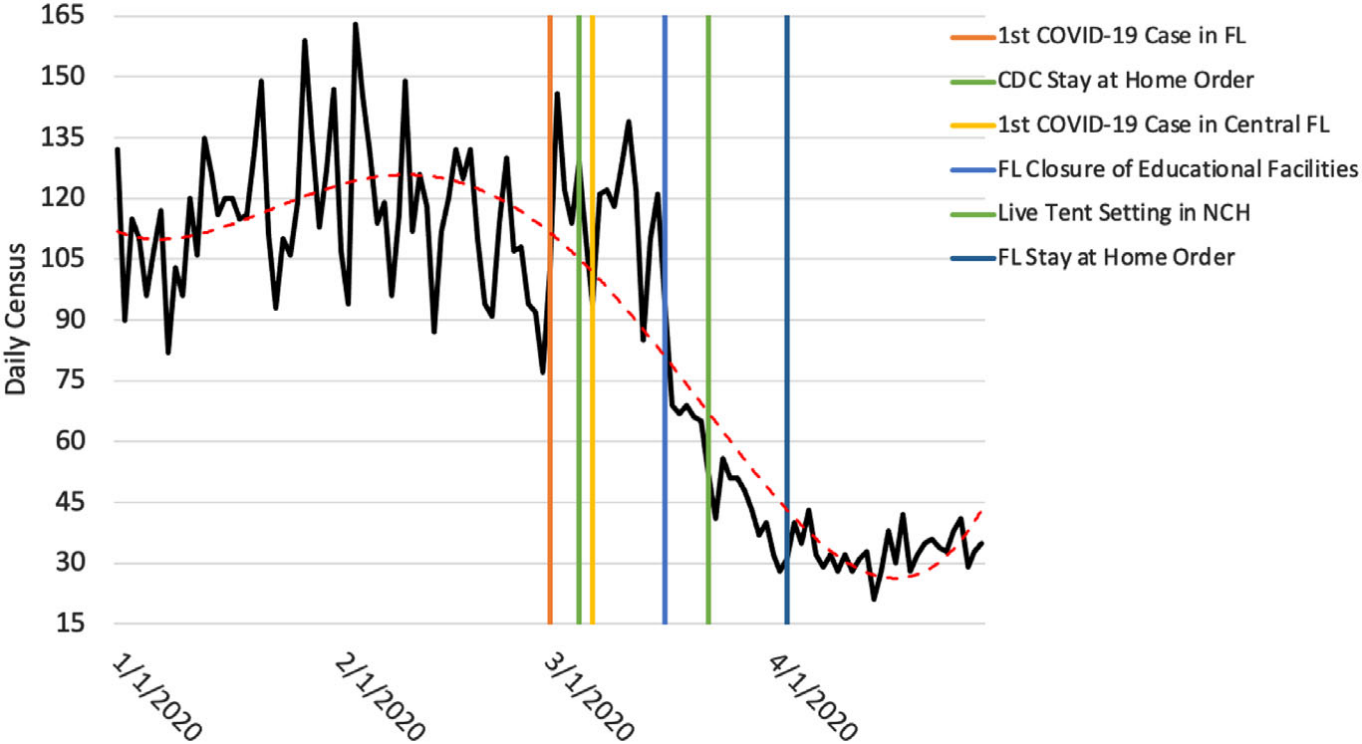
Association between daily cumulative PED census and national and state pandemic mandates by the CDC and the US government.

Temporal analysis between daily PED visits and the national pandemic mandate of the CDC’s stay-at-home order shows no discernible effect in the patient distribution (Figure 2). However, from the date of the Florida state mandate for the closure of educational facilities in mid-March 2020, there was a sharp decrease in daily PED census (from 3.4% to 1.9%), with a decreasing pattern that persisted through the Florida stay-at-home order and the rest of the study period.

## Discussion

The COVID-19 pandemic has presented challenges to the health-care system and the delivery of medical care during its early stages. The medical community has found itself attempting to anticipate the needs of their respective populations with limited information about the disease’s epidemiology, pathophysiology, morbidity, and mortality, while allocating necessary resources and protective measures. Our study shows our initial response to the COVID-19 pandemic in the PED, and how it affected the flow of patient visits and resource use. According to the CDC, ED visits across the country were down 42% in late March and April 2020 compared with the same period a year earlier.^9^ ED visits declined for every age group, with the largest proportional declines in visits by children aged ≤10 y (72%) and 11-14 y (71%).^9^ In comparison, we found a decrease in PED visits of 24% in late March and 71% in April 2020 across study periods, but no difference in patient age. The decrease in PED visits during the COVID-19 pandemic differs from the literature on the rates of ED visits and patient behavior in prior epidemics,^6^ which suggests that PED use varies depending on population-specific morbidity.

The analysis between the COVID-19 pandemic and the 2017-2018 influenza epidemic showed that influenza activity in the United States during the 2017-2018 season began to increase in November, reaching high activity during January and February nationally, and remained elevated through the end of March.^10,11^ These changes in PED visits were reflected in our 2018 analysis for the months of January and February. By comparison, there was a lower PED visit rate in March and April 2020, which may suggest changes in PED visit use related to the beginning stages of the COVID-19 pandemic. The distribution of primary diagnoses upon disposition during the study period was statistically different for each month. The CDC reported that the proportion of infectious disease-related visits was 4 times higher during the early pandemic period of late March and April 2020.^9^ Our study showed that the category of infectious cases saw an increase during the month of March 2020; whereas respiratory cases, including the primary diagnosis of influenza, were seen to decrease during the month of April 2020. This differs from the literature where respiratory conditions follow a strong seasonal variation with higher volumes beginning in October and extending through March.^12^

While it is possible that a re-categorization of such cases could have yielded different results, a sub-group analysis that included influenza and influenza-like illness as a primary diagnosis did not show any significance for the months of March or April across study periods. Because the codification of primary diagnoses was limited to 10 categories, which were used to analyze >1000 unique diagnoses, it is difficult to ascertain whether our findings represent a significant departure from baseline; therefore, further analysis is required. CDC data demonstrated that, among children aged ≤10 y, the largest declines in visits during the COVID-19 pandemic were for influenza (97% decline), otitis media (85%), other specified upper respiratory conditions (84%), nausea and vomiting (84%), asthma (84%), viral infection (79%), respiratory signs and symptoms (78%), abdominal pain and other digestive or abdomen symptoms (78%), and fever (72%).^9^ These declines may be due to statewide stay-at-home and social distancing mandates, thereby decreasing the dissemination of infectious processes, including both COVID-19 and noncoronavirus infections that normally drive ED visits. Furthermore, it may also decrease risky behavior resulting in fewer injuries; however, our study showed an increase in injuries during the month of April 2020, most likely due to an increase in outside activities.

A decline in PED visits may indicate that people may have been managing certain medical issues at home, the primary care or urgent care setting, rather than risk a hospital visit; and, therefore, avoiding hospitals due to COVID-19 fear, despite needing immediate treatment.^13^ Fluctuations in PED visit rates may demonstrate seasonal variation, but certain diagnoses, such as those surgical in nature, should continuously present at a steady rate.^14^ An example is the evaluation of abdominal pain for surgical conditions, of which our study showed a significant decrease in March 2020. Furthermore, we found no significance in surgical cases across study periods for March or April 2020. Admissions to the surgery service, however, increased in January and March 2020, but decreased in April. This may be explained by patients requiring surgery with admission to other services for management and stabilization. Also, cases with a primary diagnosis of abdominal pain may have an infectious trigger in origin, such as the case of adenovirus causing lymphoid hyperplasia in cases of acute appendicitis,^15^ which would explain their decreased prevalence during the early stages of the pandemic.

Our study showed that only PED daily census and the ESI were significantly different across study periods. The year 2020 showed a relative increase in cases classified as ESI 5, with concurrent decrease in ESI 2-4. An increase in ESI 5 cases may have been driven by a decreased availability of primary care in the community. However, there was a strong relationship between the ESI 3 and ICU admission, with a 2% increase, representing over 21,000 cases. This may suggest a higher level of acuity, not initially identified by resource requirement upon arrival to the PED. Cases classified with an ESI 3 may also appear to be less sick than an ESI 2, but may be more complex, hence, the need for admission.

The discharge rate from the PED was 92%, with 7.9% of PED visits resulting in admissions. An analysis performed by McDermott et al.^12^ on the evaluation of PED visits by the Agency for Healthcare Research and Quality found that, in 2015, 97% of PED visits were discharged home, with 3.3% admissions. The perceived deviation of the discharge and admission rate in our population compared with the national rate may be explained by a relative increase in disease severity or higher acuity at presentation, as exemplified by the month of April 2020 when an admission increase from 8.0% to 14% was noted. This could be elucidated by further study, focusing on specific diagnoses and markers of acuity. Another reason may be that the composition of the patient population using this specialty-based, free-standing children’s hospital has a more complex medical history, which may in turn reflect a higher rate of admission for resource use. Furthermore, the analysis of admissions in January 2020 moves toward a direction that may suggest a significant trend which is not reached; therefore, more in-depth analysis of this sub-group may be needed to determine statistical significance.

There was a decrease in PED visits and PED-LOS. These may be linked, but changes in protective practices and other processes to address the pandemic should be explored. A nurse-driven protocol was used in a PED tent system to triage and perform medical screening examinations to recognize and effectively manage patients with influenza-like illness or fever who were suspected of having a COVID-19 infection. PED tent turnaround time was 55-80% shorter compared with the PED flow metrics during the months of March and April 2020. The implementation of a rapid screening process, such as the PED tent system, with disregard to the volume of PED visits was associated with improved patient flow without affecting rates of return to the ED within 48 h. Although these changes were made to maximize patient recovery and outcomes while protecting the hospital personnel and the community, further study is needed to address the effects that the implementation of this strategy had on personnel acquiring a COVID-19 infection, staffing, and cost-effectiveness for future re-implementation processes.

In response to the 2017-2018 influenza epidemic, our hospital designed a volume-response system, with added additional clinical space with overflow rooms, staffed primarily by ED staff but also with a pool of people that were emergently credentialed to see patients in the ED as needed. While we had a similar contingency plan available for the ED and hospital in response to COVID-19, the system we developed and implemented addressed an isolation need. We set up an isolation system designed to keep patients who might be potentially affected outside of the physical footprint of the ED and away from the hospital and other staff. This system relied primarily on the ED staff, but backup plans to use other staff in the hospital were put in place to be used if necessary. Our experience leads us to conclude that rapid analysis of epidemiologic characteristics in future pandemics may lead to dramatically different staffing and process models driven by transmissibility and acuity of impact in specific demographic groups.

The pattern of PED visits suggests an association with state pandemic mandates, rather than national mandates, but a statistical relationship was not established. Because there might be a complex relationship between social, economic, geographic, and psychological variables, we were unable to establish cause and effect. This may suggest, however, that people within specific states may be more likely to follow their respective, state-driven mandates, rather than follow nationwide recommendations. Further study is needed to elucidate these findings.

Ultimately, the earliest interventions and guidance from the federal or state level had limited impact on the hospital’s experience of and initial response to the COVID-19 pandemic. However, in contrast to the volume surge seen in the 2017-2018 influenza epidemic, it is clear that subsequent state-required isolation and public health measures played a significant role in curtailing the expected, but not seen, volume surge during the early COVID-19 pandemic. The 2017-2018 influenza epidemic public health efforts were focused on vaccination, and its hospital response was based on volume. During the COVID-19 pandemic, the public health measures at the state and federal levels led to a decrease in the spread of the disease among the public, especially children, and allowed for hospital resources to focus efforts on isolation.

As COVID-19 continues to spread and become endemic, time will tell how the population responds to the nationwide vaccination efforts against the virus, the exposure to new variants, and their epidemiologic behavior. Furthermore, it is clear that deployment of public health measures addressing respiratory infectious diseases (that is physical measures such as hand hygiene, mask-wearing, and social distancing) limit the transmission of illness. In the future, the extent of implementation of such measures will determine the degree to which mitigation of disease spread is successful. In public health events, we can implement the preventive strategies that address both volume mitigation and isolation needs ahead of time to facilitate development of processes and allocation of staff and other resources in an appropriately responsive manner.

### Limitations

This study sought to describe the use and flow of a single PED during the early stages of the COVID-19 pandemic, which may affect the generalizability of the study. Albeit, the limited literature on the topic has shown similar findings in other children’s hospitals. We used a database of patients seen in the PED as part of the quality metrics of standard of care and not medical charts; therefore, the lack of individual patient characteristics prevents us from performing further analysis. Because PEDs are required to stabilize and treat patients regardless of their age or insurance status, the adult population was not excluded from the study. Patients who were ≥18 y of age accounted for 1.1% of total PED visits, and posed no significant deviation in the analysis. Diagnostic categories rely on the use of prioritized diagnoses and might be used inconsistently across providers, which could result in misclassification. Cases may have been miss-categorized, which in turn may have led to an underestimation or overestimation of the frequency of certain cases/categories. Finally, because this analysis is limited to PED visit data, the proportion of persons who did not visit the PED but received treatment elsewhere is not captured.

## Conclusions

The early stages of the COVID-19 pandemic affected negatively patient flow and use of the PED; with a decrease in PED visits and PED-LOS. There was an increase in admission rate that may be related to higher acuity at presentation. Our study shows that the use of the PED during a pandemic may be directly related to the effect the virus may have on a specific population. The implementation of a PED tent system helped offset the use of resources within the PED, as well as decrease unnecessary exposure to medical personnel. To prepare for potential changes in patient flow within the PED in times of public health emergencies; first, it is imperative to understand the epidemiology, pathophysiology, morbidity, and mortality of the infection before the allocation of resources. Second, to reassure anxious patients and their families about the need to visit the PED by following CDC infection control guidelines to prevent COVID-19 transmission.^16,17^ And third, changes must be made to the PED flow to better serve the needs of the hospital, personnel, and patient population. By understanding the interaction between hospital processes on PEDs and patient factors during a pandemic, and other public health emergencies, we are able to anticipate and better allocate resources in the future.
